# Mitochondria as novel mediators linking gut microbiota to atherosclerosis that is ameliorated by herbal medicine: A review

**DOI:** 10.3389/fphar.2023.1082817

**Published:** 2023-01-17

**Authors:** Yujuan Li, Shengjie Yang, Xiao Jin, Dan Li, Jing Lu, Xinyue Wang, Min Wu

**Affiliations:** ^1^ Guang’an Men Hospital, China Academy of Chinese Medical Sciences, Beijing, China; ^2^ Beijing University of Chinese Medicine, Beijing, China

**Keywords:** atherosclerosis, mitochondria, gut microbiota, metabolism, herbal medicine

## Abstract

Atherosclerosis (AS) is the main cause of cardiovascular disease (CVD) and is characterized by endothelial damage, lipid deposition, and chronic inflammation. Gut microbiota plays an important role in the occurrence and development of AS by regulating host metabolism and immunity. As human mitochondria evolved from primordial bacteria have homologous characteristics, they are attacked by microbial pathogens as target organelles, thus contributing to energy metabolism disorders, oxidative stress, and apoptosis. Therefore, mitochondria may be a key mediator of intestinal microbiota disorders and AS aggravation. Microbial metabolites, such as short-chain fatty acids, trimethylamine, hydrogen sulfide, and bile acids, also affect mitochondrial function, including mtDNA mutation, oxidative stress, and mitophagy, promoting low-grade inflammation. This further damages cellular homeostasis and the balance of innate immunity, aggravating AS. Herbal medicines and their monomers can effectively ameliorate the intestinal flora and their metabolites, improve mitochondrial function, and inhibit atherosclerotic plaques. This review focuses on the interaction between gut microbiota and mitochondria in AS and explores a therapeutic strategy for restoring mitochondrial function and intestinal microbiota disorders using herbal medicines, aiming to provide new insights for the prevention and treatment of AS.

## 1 Introduction

Atherosclerosis (AS) is an inflammatory disease characterized by endothelial cell damage, lipid deposition, and smooth muscle cell proliferation. It is one of the leading causes of cardiovascular disease (CVD) and is responsible for morbidity and mortality worldwide (Cai et al., 2020; Dagenais et al., 2020). Recent studies have focused on the intestinal flora and mitochondrial dysfunction as risk factors for AS (Witkowski et al., 2020). In the distal gastrointestinal tract, gut flora plays an important role in host physiology by establishing bacterial–host symbiosis, whereas its imbalance regulates host inflammation and metabolic and immune disorders, triggering the development of AS (Barrington and Lusis, 2017; Jonsson and Bäckhed, 2017). Moreover, bacterial translocation and metabolites entering circulation aggravate atherosclerotic plaque severity by directly invading arteries, increasing lipid deposition and insulin resistance, and activating the innate immune system (Witkowski et al., 2020). The predominant intestinal flora metabolites include short-chain fatty acids (SCFAs), trimethylamine (TMA), amino acids and their derivatives, secondary bile acids, and vitamins, all of which are associated with AS (Koeth et al., 2013). Although the mechanism and relationship between the gut flora and AS are complex, mitochondria could be an intermediate link, which might explain why AS occurs locally. As the center of cellular aerobic respiration, mitochondria synthesize 90% adenosine triphosphate (ATP) to provide the energy required by cells (Rossmann et al., 2021). Owing to an endosymbiotic relationship with ancient proteobacteria (Roger et al., 2017), mitochondria are also responsible for the signal transduction of innate immunity, oxidative stress, and cell death (Forrester et al., 2018). Thus, mitochondria are often targeted for bacterial attack and show functional abnormalities, including mitochondrial reactive oxygen species (mtROS)-induced oxidative stress, mitochondrial DNA (mtDNA) mutation and release, and imbalance of mitochondrial dynamics, further contributing to arterial wall cell damage, inflammation, and AS (Yu and Bennett, 2014; Oliveira and Vercesi, 2020).

Current treatments for AS mainly aim at improving known risk factors. Despite significant developments in disease control and prevention, there is no clear, effective treatment for these novel mechanisms (Pursnani et al., 2017; Lawton et al., 2022). Herbal medicines have been widely used to treat AS, and a possible therapeutic target is to regulate gut microbiota homeostasis, which is regarded as prebiotics (Anlu et al., 2019). Herbal medicines and their natural compounds can effectively improve mitochondrial function to protect vascular cell homeostasis and survival (Zhou et al., 2022). In this review, we discussed the emerging mechanisms of mitochondria as the key mediator linking gut microbiota to AS and systematically reviewed the role of herbal medicine and their natural compounds in improving AS through the microbiota–mitochondrial axis.

## 2 Vital role of mitochondria in AS

### 2.1 Mitochondrial dysfunction

During AS progression, mitochondrial dysfunction is associated with progressive deleterious changes, including 1) the accumulation of mtDNA mutations, 2) excessive production of mtROS and diminished antioxidant defense, leading to increased oxidative damage, and 3) mitophagy inhibition, leading to a decline in mitochondrial renewal.

#### 2.1.1 mtDNA mutation

The genetic mechanisms of atherogenesis depend on the nuclear and mitochondrial genomes. In contrast, mtDNA is more vulnerable to damage from mtROS owing to its lack of introns and histones, leaving DNA exposed, and lack of DNA polymerase, leading to a high error rate and poor stability during mtDNA replication proximate to the site of ROS production (Valente et al., 2016; Stewart and Chinnery, 2021). mtDNA encodes protein subunits necessary for oxidative phosphorylation (Fontana and Gahlon, 2020), resulting in abnormal complex expression, subsequently leading to the generation of excessive mtROS vicious cycle between mtDNA mutation and mtROS (Yang et al., 2020). The other accepted reason for the occurrence of mtDNA mutations is replication errors resulting from *de novo* events and clonal expansion, and the two have an additive effect and are detrimental to overall health (Ross et al., 2014). Many somatic mtDNA mutations originate from early adulthood to clonally expand preexisting mutations (Greaves et al., 2014) and increase dramatically with age through spontaneous replication errors of mtDNA polymerase γ (Pol γ), accelerate mtDNA turnover, and direct repeats (Khrapko, 2011). The frequency of *de novo* mtDNA mutation is elevated in mouse female germ cells, being associated with age and parity or sexual maturity (Arbeithuber et al., 2020). All of these factors lead to a high mutation rate and heteroplasmy of mtDNA.

mtDNA mutations occur in the local disturbance of plaques (Sazonova et al., 2015), and the number of mtDNA mutations in the arterial wall cells increases as the lesions become more severe (Ballinger et al., 2002). mtDNA mutations, such as m.1555A>G, m.14459G>A, m.12315G>A, and m.13513G>A occur in plasma leukocytes or aortic plaques, (Sazonova et al., 2015; Sazonova et al., 2017), which may uncouple oxidative phosphorylation (OXPHOS), inactivate ATP synthase, and enhance the basal rate of oxygen consumption (Gilkerson et al., 2012; Orekhov et al., 2020b). High levels of mtDNA^4977^ deletion are independent risk factors for adverse cardiovascular events and cause mortality in patients with stable coronary heart disease (CHD) (Vecoli et al., 2018; Vecoli et al., 2019). In three cohort studies, mtDNA copy number (mtDNA-CN), which reflects normal mtDNA expression, is negatively correlated with CHD (Ashar et al., 2017; Zhang Y et al., 2017). PolG^−/−^/ApoE^−/−^ mice lacking mtDNA polymerase γ exhibit extensive mtDNA damage, OXPHOS defects, and severe AS (Yu et al., 2013). ApoE^−/−^ mice overexpressing the mitochondrial helicase Twinkle (Tw^+^/ApoE^−/−^) showed increased mtDNA-CN, respiratory complex abundance and respiration, decreased plaque necrotic core, and increased fibrous cap area (Yu et al., 2017). mtDNA mutation contribute to induction of proton leakage, transmembrane potential loss, reduced mitochondrial Ca^2+^ uptake (Granatiero et al., 2016), and activation of the adenosine monophosphate-activated protein kinase (AMPK) signaling pathway (Orekhov et al., 2020b), promoting arterial wall cell apoptosis and vascular inflammation. Consequently, mtDNA mutations contribute significantly to AS and CHD by disrupting the intracellular environment.

#### 2.1.2 Excessive production of mtROS

Mitochondrial complexes are the main producers of mtROS (Yin et al., 2021), and the excessive production of mtROS and inhibition of antioxidase cause an imbalance in redox reactions (Springo et al., 2015), which promotes mtDNA mutations (Fontana and Gahlon, 2020), aggravates NF-κB activation (Csiszar et al., 2014), excites NAD(P)H oxidase (NOX) in the cytoplasm (Canugovi et al., 2019), and develops vascular oxidative stress and inflammation. Cho et al. (Cho et al., 2013) determined that mtROS significantly increased and augmented superoxide dismutase 2 (SOD2) ubiquitination in coronary endothelial cells (ECs) from type 2 diabetic mice, leading to abnormal vascular relaxation and EC damage. mtROS attenuates nitric oxide (NO) bioavailability, impairs endothelium-dependent dilation, and uncouples nitric oxide synthase (eNOS), contributing to vasodilation inhibition (Robert et al., 2021). Apoe^−/−^/SOD2^+/−^ mice treated with MitoTEMPO targeting mtROS showed reduced smooth muscle cell apoptosis, necrotic core expansion, and matrix degradation to stabilize plaques (Vendrov et al., 2017). Mitochondria-derived H_2_O_2_ induces NF-κB activation in aged endothelial cells, which elevates low-grade vascular inflammation, such as increased levels of proinflammatory cytokines and adhesion molecules (Li Z et al., 2021). Mitochondrial H_2_O_2_ also induces endothelial-to-mesenchymal transition (EndoMT) *via* the p38 MAPK or NF-κB signaling pathways to cause vascular dysfunction and AS (Ko et al., 2019; Xu et al., 2020). EndoMT is a dynamic process that loses characteristic endothelial tight junctions and markers, including CD31, VE-cadherin, and eNOS, and acquires the mesenchymal phenotype (Xu et al., 2020). Administration of natural compounds, such as Baicalein (Shi R et al., 2018) and schizandrin B (You et al., 2019), attenuates EndoMT by suppressing the NF-κB pathway. Mitochondria-targeted antioxidants, including mitoquinone (MitoQ), MitoTEMPO, and coenzyme Q 10 (CoQ10) effectively ameliorate AS (Rossman et al., 2018). MitoQ intervention inhibits nitrotyrosine concentration and improves mitochondrial and endothelial functions (Braakhuis et al., 2018). MitoQ also decreases aortic stiffness in old mice by weakening the aortic pulse wave velocity and restoring the elastin region elastic modulus and elastin expression (Gioscia-Ryan et al., 2018). CoQ10 treatment of AS attenuates mtROS generation and activates the AMPK–YAP–OPA1 pathway to improve mitochondrial function (Xie et al., 2020). Therefore, mitochondria-derived ROS are key risk factors for cell damage, inflammation, and AS. Decreased mtROS production effectively mitigates atherosclerotic lesions.

#### 2.1.3 Inhibition of mitophagy

Mitophagy is a selective type of autophagy that acts by removing unnecessary or aberrant mitochondria and its harmful metabolites, such as mtROS, ensuring mitochondrial quality control and stabilizing cell homeostasis (Kubli and Gustafsson, 2012). Autophagy can be divided into ubiquitin-dependent mitophagy and receptor-regulated mitophagy (Song et al., 2021). Mitophagy is inhibited in plaque lesions. Under high-glucose conditions, putative kinase 1 (PINK)/Parkin acetylation is defective in aortas regulated by sirtuin 3 (SIRT3) or uncoupling protein 2 (UCP2) (Wei et al., 2017), triggering impaired endothelial cells and aggravating AS by excessive mtROS, loss of mitochondrial membrane potential (MMP), and ATP reduction (Zhu W et al., 2018; Forte et al., 2021). Deficiencies in PINK1 or Parkin proteins result in vascular smooth muscle cell (VSMC) apoptosis by inhibiting mitophagy (Swiader et al., 2016) and increasing the occurrence of myocardial infarction by the accumulation of swollen and dysfunctional mitochondria (Kubli et al., 2013). Activation of the PINK1/Parkin pathway maintains mitochondrial integrity and avoids damage to the endothelial cells of obese mice (Wu W et al., 2015), leading to reduced apoptosis by mitigating B Cell lymphoma-associated X (BAX). Apolipoprotein A–I binding protein (AIBP) located at the inner mitochondrial membrane (IMM) is expressed in human and mouse atherosclerotic plaques (Choi et al., 2021). AIBP regulates PINK/parkin-related mitophagy and stimulates mitofusin 1 (MFN1) and MFN2 ubiquitination, improving mitophagy and fission to prevent further cell damage in AS. Therefore, enhanced mitophagy is beneficial for diminishing apoptosis, restoring endothelial function, and protecting the vasculature from sclerosis. In conclusion, the effects of mitochondrial dysfunction on AS have gradually become a focus of research. Injury to mtDNA, mtROS, and mitophagy are all involved in inflammation, disruption of cell homeostasis, and the development of AS.

### 2.2 Mitochondrial regulation of innate immunity and inflammation

Mitochondria are mostly known for immunity, inflammation, and apoptosis. For example, mitochondrial energy supply depends on the tricarboxylic acid cycle, and its disruption generates metabolites involved in the inflammatory response and modulates the innate immune response in immune cells (Banoth and Cassel, 2018). Downregulation of isocitrate dehydrogenase leads to the conversion of citrate to itaconate (Lampropoulou et al., 2016), which has direct antimicrobial characteristics that stimulate the innate immune response and enhance the proinflammatory properties of M1 (Peace and O'Neill, 2022). Succinate is also proinflammatory and induces mtROS production and IL-1β expression (Banoth and Cassel, 2018).

#### 2.2.1 Mitochondrial damage-associated molecular patterns

As mitochondria evolved from ancient eubacteria, mtDNA contains hypomethylated cytosine-phosphate-guanine sequences similar to those in bacterial DNA (Fang et al., 2016). In addition to mtDNA, mitochondria-derived DAMPs (mtDAMPs) also include other mitochondrial metabolites, such as mtROS, phospholipid cardiolipin, and formyl peptides, which can be recognized as pathogens, elicit inflammation, and induce an innate immune response (Zhang et al., 2010). They are released from impaired mitochondria and activate formyl peptide receptor or pattern recognition proteins (PRPs), which respond to multiple inflammatory signaling pathways, including the nod-like receptor family pyrin domain containing 3 (NLRP3) inflammasome, NF-κB activation, and stimulator of interferon genes (STING) pathways (Misawa et al., 2017). Therefore, mitochondria are crucial elements in sterile inflammation, leading to chronic inflammation in AS.

Mitochondrial permeability transition pores (mPTP) and voltage-dependent anion channel (VDAC) oligomers are responsible for mtDAMP release (Sprenger et al., 2021; Picca et al., 2022). mPTP opening occurs in cells subjected to disrupted calcium levels or oxidative stress (Kim et al., 2019). Binding of mtDNA with charged residues in the N-terminal domain of VDAC1 facilitates VDAC1 oligomerization, which is instrumental in the release of mtDNA fragments over the outer membrane and increases outer membrane permeabilization mediated by BAX and B Cell lymphoma antagonist/killer (BAK) (McArthur et al., 2018). In addition to releasing large molecules from the mitochondria, VDAC also affects ROS production. When VDAC is inhibited by hexokinase, mtROS expression and NLRP3 activation are downregulated (Kim et al., 2019; Xian et al., 2022). Ultimately, mtDAMP, in the form of mitochondrial-derived extracellular vesicles, crosses the cytomembrane (Zhao et al., 2021).

#### 2.2.2 mtDAMPs activate NLRP3 inflammasome

NLRP3 inflammasome is a protein complex of the NLR family. Activated NLRP3 is regulated by the adaptors of damaged mitochondria and endoplasmic reticulum (ER), further inducing caspase-1 expression and promoting interleukin (IL)-1β and IL-18 secretion, further causing inflammation and pyroptosis (Zhou et al., 2011; Song et al., 2020), which can be demonstrated by the colocalization of mitochondria with NLRP3–ASC–caspase–1 (Mohanty et al., 2019). Oxidative stress is a proinflammatory characteristic of impaired mitochondria (Heid et al., 2013). Oxidized mtDNA and mtROS interact with NLRP3 on being released into the cytoplasm (Xian et al., 2022). Depletion of mtDNA using ethidium bromide or knockout of mitochondrial transcription factor A (TFAM) suppresses NLRP3 activation and IL-1β expression (Salnikova et al., 2021).

Mitochondria also provide other key signals to recruit and activate the NLRP3 inflammasome, including translocation of cardiolipin, mitochondrial antiviral signaling proteins (MAVS), and cytosolic potassium ion (K^+^) efflux (Bird, 2012). Cardiolipin plays a key role in triggering NLRP3 owing to its role as a relic of the prokaryotic cell membrane (Iyer et al., 2013). Cardiolipin is located in the IMM and is transferred to the outer mitochondrial membrane (OMM) after depolarization to recruit NLRP3 (Gkikas et al., 2018). After knockout of cardiolipin synthase to inhibit cardiolipin, the NLRP3 inflammasome is effectively downregulated, and the inflammatory response is attenuated, indicating that cardiolipin triggers NLRP3 (Toksoy et al., 2017). MAVS, located in the OMM, is a critical signal for antiviral infection. MAVS is also involved in NLRP3 activation (Subramanian et al., 2013). Specifically, it recruits NLRP3 to the OMM and mediates its oligomerization and activation by targeting mtROS (Park et al., 2013). MAVS is a key sensor of mtROS, contributing to the production of type I interferon (IFN) and activation of caspase-1, and is responsible for host immunity and inflammation (Buskiewicz et al., 2016). Furthermore, ATP and K^+^ efflux are regarded as two key NLRP3 activators caused by impaired mitochondria and induce Ca^2+^ influx and direct damage to mitochondrial Ca^2+^ homeostasis (Yaron et al., 2015; Katsnelson et al., 2016), further affecting Krebs cycle enzyme activity, leading to a loss of MMP and mitochondrial network fragmentation (Hawkins et al., 2007; Liu et al., 2018). Mitochondrial Ca^2+^ homeostasis plays a crucial role in plaque calcification and unstable lesions in AS (Forrester et al., 2018).

Mitophagy mediates innate immunity and prevents hyperstimulation of NLRP3 and superfluous inflammation (Orekhov et al., 2020b). As defective mitophagy constantly emits inflammatory signals, cells cannot bear the ongoing stress and undergo apoptosis (Jin et al., 2022). Melatonin inhibits mtROS expression and suppresses NLRP3 inflammasome activation by enhancing mitophagy in lipopolysaccharide-treated (LPS)-treated macrophages, thereby preventing AS progression (Ma S et al., 2018). Accordingly, NLRP3 may be sensitive to mitochondrial dysfunction, explaining the correlation between mitochondrial dysfunction and chronic inflammatory diseases. Furthermore, NLRP3 exacerbates mitochondrial dysfunction. For example, caspase-1 induced by NLRP3 enhances mitochondrial membrane permeabilization and mtROS generation, and NLRP3 disturbs mitochondrial Ca^2+^ homeostasis (Yu et al., 2014). These suggest a positive feedback loop between NLRP3 and mitochondria (Orekhov et al., 2020a).

#### 2.2.3 mtDAMPs induce other inflammatory pathways

Cyclic guanosine monophosphate–adenosine monophosphate synthase (cGAS) effectively senses and recognizes viral and bacterial DNA in the cytoplasm. Released mtDNA is also an endogenous cGAS ligand that binds to cGAS and catalyzes the generation of cyclic guanosine monophosphate–adenosine monophosphate (cGAMP) (Chowdhury et al., 2022). cGAMP is a secondary messenger that recruits STING (Yu et al., 2020), which stimulates tank-binding kinase in perinuclear endosomes to mediate the phosphorylation of the transcription factor interferon regulatory factor 3 (IRF-3) and transposition into the nucleus (West et al., 2015), promoting type I and III IFN responses and interferon-stimulated gene activation (Puhm et al., 2019). cGAMP expression is elevated in the arteries of ApoE^−/−^ mice, stimulating the accumulation of lipids and macrophages and the secretion of inflammatory cytokines (Pham et al., 2021). However, STING deficiency prevents the progression of atherosclerotic plaques by inhibiting inflammation (Pham et al., 2021). Thus, the cGAS–STING pathway enhances vascular inflammation induced by mtDNA to aggravate AS.

Mitochondria engage in antimicrobial processes of toll-like receptor (TLR) signaling cascades. Activated TLR send signals to the mitochondria by translocating tumor necrosis factor receptor associated factor 6 (TRAF6) and ubiquitinate the evolutionarily conserved mitochondrial signaling intermediate in toll pathways (ECSIT) to stimulate mtROS production and move mitochondria to phagosomes, enhancing antimicrobial activity (West et al., 2011). Depletion of ECSIT and TRAF6 decreases the expression of mtROS and attenuates antimicrobial ability (Banoth and Cassel, 2018). TLR9 is an innate immune receptor that recognizes CpG motifs in bacterial or viral DNA and releases mtDNA (Hotz et al., 2018). Extracellular mtDNA triggers NF-κB nuclearization, induces proinflammatory cytokines, augments type I IFN responses, and provokes a sterile systemic inflammatory response syndrome mediated by TLR9 activation (Zhang et al., 2014; Bliksøen et al., 2016). Circulating oxidative mtDNA triggers TLR9 activation and upregulates proinflammatory cytokines in macrophages of ApoE^−/−^ mice undergoing e-cigarette intervention, which leads to AS. Inhibition of the inflammatory cascade depends on normal mitophagy (Li J et al., 2021). Mitophagy is suppressed *via* PINK degradation, promoting macrophage polarization of the proinflammatory phenotype M1 and the development of AS (Hung et al., 2021). Consequently, TLRs are recognized and stimulated by mitochondria-derived DAMPs, such as mtDNA, and further trigger amplification of the inflammatory cascade to exacerbate AS progression.

### 2.3 Mitochondrial regulation in vascular cells

Various cells play different roles in AS progression, including barrier protection function of ECs, vasoconstriction and remodeling function of VSMCs, phagocytosis of lipids and immune function in macrophages, with mitochondria participating in all.

#### 2.3.1 Cholesterol metabolism in macrophages

Mitochondria play a crucial role in lipid metabolism during AS progression. Modified low-density lipoprotein (LDL) is phagocytosed by macrophages and focally stimulates vascular innate immunity, where the mitochondria-associated immune barrier is compromised, contributing to lipid deposition, accumulation of proinflammatory mediators, and transformation of lipid-rich foam cells (Duan et al., 2022). In ECs, intervention with oxidized LDL (ox-LDL) causes mtROS significantly increase, reduction in complex I and III enzyme activity, mPTP opening, cytosolic Ca^2+^ influx, promoting mitochondrial damage and cell apoptosis, triggering vascular inflammation, and the occurrence of AS (Orekhov et al., 2020b). Mitochondrial respiratory dysfunction negatively influences cholesterol efflux in macrophages. Reverse cholesterol transport (RCT) is mediated by ATP-binding cassette cholesterol transporter proteins (ABCA1) and apolipoprotein A–I (ApoAI) in macrophages, leading to the accumulation of cholesterol and an increase in foam cells (Dumont et al., 2021; Dorighello et al., 2022). ATP is the energy provider for ABCA1-dependent cholesterol efflux. miR-33 also controls cholesterol efflux by limiting ATP production (Karunakaran et al., 2015). Excessive accumulation of cholesterol stimulates mPTP to open and enter the mitochondria, subsequently generating multiple oxysterols *via* CYP27A1 (a cytochrome p450 oxidase), such as 25- and 27-hydroxycholesterol (25-/27-OH) in the mitochondria, and constantly esterifies oxysterol in atherosclerotic plaques (Korytowski et al., 2015). Cholesterol oxides inhibit ABCA1 and ABCG1 expression and diminish 27-OH efflux inside the mitochondria of macrophages, leading to RCT impairment and peroxidation damage in the mitochondria (Karunakaran et al., 2015). AIBP binds to ApoAI and induces mitophagy and NADPH, whereas a deficiency in AIBP leads to the accumulation of damaged mitochondria and polarization in M1 macrophages to promote inflammation. Furthermore, interventions targeting the mitochondria can recover cholesterol trafficking in AS. Mitochondrial antioxidant Mito-Tempol attenuates cholesteryl accumulation by activating ABCA1 expression to export cholesterol (Ma Y et al., 2018). Pravastatin is involved in mitochondrial fusion and the formation of mitochondria-associated membranes to strengthen the correlation between mitochondria and the ER, further decreasing foam cell generation in a high-lipid environment (Assis et al., 2022). Therefore, recovering mitochondrial respiratory function and providing efficient energy ameliorates cholesterol trafficking, decreases foam cell numbers, and reverses AS progression.

#### 2.3.2 VSMC proliferation, migration, and phenotype switching

VSMC proliferation, migration, and phenotype switching are critical features of AS and contribute to intimal hyperplasia and restenosis in the arteries. VSMC exhibit a contractile phenotype that modulates vascular dilation and contraction under physiological conditions. After vascular injury from various stresses, VSMC convert to a synthetic phenotype, minimizing contractile function and migrating and proliferating from the medial layer to the arterial lumen (Chen et al., 2022). VSMC phenotype switching is closely associated with mitochondrial functions, including fission, oxidative stress, mitophagy, and Ca^2+^ influx. Mitochondrial fission regulates the quantity and quality of mitochondria in cells and is mediated by dynamin-related GTPases, such as dynamin-related protein 1 (Drp1) (Maimaitijiang et al., 2016). Drp1 activated by AngII, PGDF-BB, or H_2_O promotes VSMC proliferation by accelerating the cell cycle, whereas mitochondrial division inhibitor-1 (Mdivi-1) stops the cell cycle at the G0 (quiescent)/G1 (long growth) phase by inhibiting Drp1 (Lim et al., 2015), attenuating VSMC proliferation and migration (Zhang X et al., 2018). High glucose induces mitochondrial fragmentation, leading to mtROS production, oxidative stress, and VSMC proliferation, which was reversed by reducing Drp1 expression *via* Mdivi (Maimaitijiang et al., 2016) or H_2_S (Sun et al., 2016). Meanwhile, knockdown of the dominant-negative mutant Drp1-K38A or Drp1 also inhibits PDGF-induced mitochondrial fission and mtROS levels, resulting in the suppression of VSMC migration. Mitochondrial Ca^2+^ uptake by activating Ca^2+^-dependent kinase II and mitochondrial Ca^2+^ uniporter also affects mitochondrial translocation to the leading edge of migrating VSMC (Nguyen et al., 2018). Apelin-13 triggers mitochondrial Ca^2+^ influx and facilitates mtROS expression and mitophagy mediated by the PINK1/Parkin pathway, leading to the proliferation of VSMCs, whereas using mito-TEMPO, Mdivi-1, siRNA-PINK1, or siRNA-Parkin can ameliorate VSMC proliferation (Chen et al., 2022). Thus, VSMC proliferation and migration are mediated by excessive mitochondrial fission, mtROS, Ca^2+^ influx, and mitophagy, which aggravate vascular intimal remodeling and AS progression.

#### 2.3.3 Cell death

Mitochondria are strongly associated with programmed cell death, and their related mechanisms include apoptosis and pyroptosis (Bock and Tait, 2020), which is a cascade of amplified inflammatory reactions mediated by caspase-1 and inflammasome (Yu et al., 2014). Mitochondria are crucial promoters of inflammasome activation and pyroptosis, and the inhibition of mitophagy exacerbates pyroptosis. NIP3-like protein X (NIX) engages in mitophagy with microtubule-associated protein 1 light chain 3 (LC3) (Peng et al., 2020), suppresses mtROS and NLRP3 expression, inhibits caspase-1 and IL-1β activation, and induces macrophage pyroptosis in AS progression (Peng et al., 2022; Tian et al., 2022).

**FIGURE 1:**
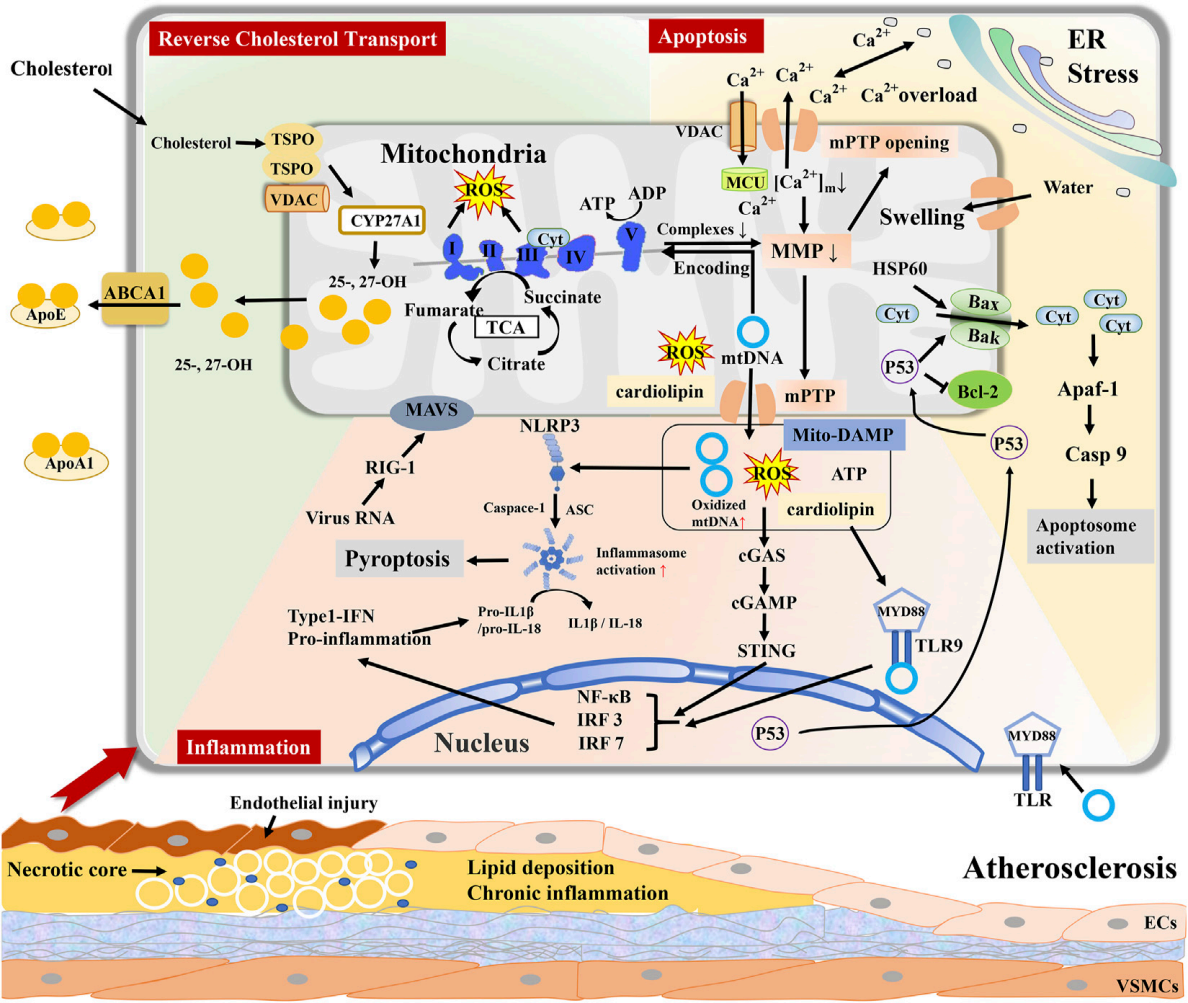
Mitochondria regulate reverse cholesterol transport (RCT), cell apoptosis, and inflammatory response during atherosclerosis. Excessive accumulation of cholesterol entry into mitochondria generate and esterify 25- and 27-hydroxycholesterol ((25-/27-OH)) by CYP27A1. Inhibition of ABCA1 results in RCT impairment and peroxidation damage in the mitochondria of macrophages. Mitochondria-DAMPs include mtDNA, mtROS, ATP, and cardiolipin, and export *via* mPTP. Mito-DAMPs are recognized as a pathogen to elicit inflammation, such as TLR9 signaling, cGAS–cGAMP–STING pathway, and inducing NLRP3 inflammasome to stimulate inflammatory cascade response. mPTP opening causes Ca^2+^ and cytochrome C (Cyt) efflux and mitochondrial swelling, leading to cellular apoptosis. Mitochondrial dysfunction contributes to lipid deposition, inflammation, and cellular apoptosis in the vascular wall, jointly promoting atherosclerosis progression.

The main mechanism of apoptosis is the enhancement of OMM permeabilization mediated by the accumulation of Ca^2+^ and activation of the proapoptotic proteins BAX and BAK (Jeong and Seol, 2008). BAX and BAK oligomerize at the OMM and for a transmembrane pore, resulting in mPTP opening, loss of MMP, and release of mitochondrial intermembrane space proteins, such as cytochrome c, apoptosis-inducing factor, and endonuclease G (Schellenberg et al., 2013). Water moves into the mitochondrial matrix *via* the opening of the mPTP, causing mitochondrial matrix swelling and OMM rupture, releasing more intermembrane space proteins into the cytoplasm and inducing apoptosis (Han et al., 2019; Liang et al., 2022). After exporting into the cytosol, cytochrome C combines with apoptotic protease-activating factor-1 and procaspase-9, composing the apoptosome structure and initiating an irreversible apoptosis cascade (Zhao Y et al., 2020). Furthermore, nitrosylation of Drp1 regulates fission and triggers the release of proapoptotic factors into the cytoplasm (Kleele et al., 2021; Jenner et al., 2022). Expression of the fusion protein optic atrophy protein 1(OPA1) suppresses apoptosis by limiting cristae remodeling, and OPA1 knockout exacerbates VSMC apoptosis and aggravates AS (Peng et al., 2020). Therefore, owing to their unique structure and function, mitochondria are responsible for the activity and death of vascular cells through the regulation of inflammation, permeability, dynamics, and activation of proapoptotic cytokines **(** Figure 1 **)**. However, the gut microbiota and its metabolites may be potential predisposing factors for these results.

## 3 Gut microbiota disorders target mitochondria and are closely correlated with AS

There are 100 trillion microbes in the human gastrointestinal tract, including 1000 different bacterial species comprising five phyla: *Bacteroidetes, Firmicutes, Proteobacteria, Actinobacteria,* and *Verrucomicrobia*, collectively regarded as “gut microbiota” (Li et al., 2014; Yoo et al., 2022). Gut microbiota and host symbiotic interactions commonly form an intestinal microenvironment to digest indigestible substances and secrete biologically active metabolites, and together maintain host energy metabolism, promote immune homeostasis, and sustain the normal function of distal organs (Gregory et al., 2015; Lian W S et al., 2022). When the intestinal flora and some of its metabolites, such as LPS, enter the bloodstream, they act as PAMP to bind to PRPs in host cells, activate immune cells, induce the production of inflammatory cytokines and chemokines necessary for protective immune responses, and present antigens to antigen-presenting cells in lymphoid tissues, resulting in systemic inflammation and excessive immune response (Verhaar et al., 2020). Meanwhile, the proliferation of opportunistic pathogens or facultative anaerobic bacteria in the intestinal tract causes gut dysbiosis and secretion of numerous abnormal metabolites, which facilitate host fat mass gain, insulin resistance, and various metabolic diseases such as obesity, diabetes, metabolic syndrome, and AS (Le Roy et al., 2022). An imbalance in the proportion of microbiota species is closely correlated with the host’s age, dietary habits, emotional stress, and antibiotic application (Boulangé et al., 2016). Thus, the gut microbiota is not only a digestive apparatus but also a dynamic endocrine organ that regulates the physiological and pathological conditions of the host (Vezza et al., 2020).

### 3.1 Gut microbiota-mediated damage to mitochondria and AS

#### 3.1.1 Flora translocation in the atherosclerotic plaque

Gut dysbiosis is strongly associated with AS and CVD, mainly through microbiota translocation into atherosclerotic plaques and microbial metabolites from the gut, which act on distal arteries to affect AS progression (Lindskog Jonsson et al., 2017). DNA from *Bacteroides forsythus, Prevotella intermedia, Porphyromonas gingivalis, genus Curvibacter,* and *Actinobacillus actinomycetem comitans* have been detected in human endarterectomy plaques (Koren et al., 2011; Ziganshina et al., 2016). *Burkholderiales* and *Propionibacterium* are the most abundant taxa in atherosclerotic plaques (Ziganshina et al., 2016). Several structural components of the gut microbiota directly stimulate the expression of proinflammatory cytokines and chemokines, such as LPS, peptidoglycan, flagellin, and bacterial DNA. These bacteria that invade arteries not only contribute to local infections and inflammation but also aggravate the total cholesterol and fibrinogen levels to exacerbate atherosclerotic plaque ruptures (Brown and Hazen, 2018).

Gut bacterial abundance and diversity are altered in ApoE^−/−^mice with AS, including decreased α-diversity (Kappel et al., 2020) and increased abundance of *Verrucomicrobia,* Bacteroidaceae*, Bacteroides, Akkermansia,* and *phylum Actinobacteria*, which positively correlate with serum cholesterol, triglycerides, and LDL levels, inducing TLR signaling and cytokine receptor interactions (Liu Q et al., 2020). Similar alterations in the gastrointestinal tract of human atherosclerotic patients have also been found, such as enrichment in *Collinsella* or *phylum Actinobacteria*, which increase gut permeability and triglyceride synthesis, recruit neutrophils, and trigger the NF-κB pathway (Chen J et al., 2016; Gomez-Arango et al., 2018). In contrast, probiotics are involved in multiple key processes of AS development by attenuating intestinal flora disorders and the risk of infection (Zhai et al., 2022). L*actobacillus strains,* such as *Lactobacillus rhamnosus JL1* (Yang et al., 2021)*, Lactobacillus pentosu*s (Huang et al., 2014)*,* and *Lactobacillus acidophilus* (Michael et al., 2017 *),* decrease total cholesterol, triglyceride, and LDL levels in mice fed a high-fat diet. *Lactobacillus* c*fermentum* effectively protects endothelial function and prevents oxidative stress by maintaining intestinal homeostasis and inhibiting eNOS uncoupling and NOX2 activation (Toral et al., 2018). Moreover, *Bifidobacterium* ameliorates inflammatory factors, such as TNF-α and IL-6, resulting in the reversal of AS progression (Primec et al., 2019; Zaharuddin et al., 2019).

#### 3.1.2 Mitochondrial injury from gut microbiota

The correlation between the gut microbiota and mitochondria can be traced back to α-Proteobacteria as the origin of the mitochondria (Roger et al., 2017). Mitochondria have homologous characteristics with bacteria; therefore, bacterial and mitochondrial protein-targeting sequences exhibit similarities and close lineage, leading to mitochondria becoming a target for gut microbiota (Degli Esposti et al., 2014). Similar autophagic methods eliminate both mitochondria and bacterial membranes, whereas abnormal autophagy contributes to chronic inflammation by inducing cytokine and inflammasome expression in the host (Kalghatgi et al., 2013). Consequently, mitochondria-related innate immunity activation is an important target of the gut microbiota (Jackson and Theiss, 2020). *Clostridium difficile* toxin A triggers mtROS production and ATP dissipation, which are associated with upregulation of NF-κB and IL-8 (He et al., 2002). *Salmonella enterica* serovar *Typhimurium* stimulates macrophage activation and induces an innate immune response by releasing mtDNA into the cytosol to induce a type I IFN response and the cGAS–STING pathway (Xu L et al., 2022). During these processes, gut bacterial effector proteins transit to the mitochondrial subcompartments *via* complex import systems and are precisely located in the mitochondria by targeting the sequence domain (Mahmud et al., 2021). Specifically, bacterial effector proteins are recognized and translocated by the translocase of the OMM complex and transported to the OMM, mediated by the sorting and assembly machinery complex. The effector proteins are subsequently transferred into the IMM and matrix by the translocase of the inner membrane 22 (TIM22) and TIM23 complexes, resulting in mitochondrial dysfunction and risk of bacterial survival and infectivity (Mahmud et al., 2021).

Furthermore, as mitochondria are organelles that regulate apoptosis, the consequences of infection from the gut microbiota usually target mitochondria-mediated apoptosis to enhance its virulence. enteropathogenic *E coli* produces the cytotoxins Maps EspZ and EspF, precisely targeting the mitochondria. EspF induces apoptosis *via* the release of cytochrome c from the mitochondria, whereas EspZ inhibits apoptosis to sustain MMP (Shames et al., 2011). Maps are also transported to the mitochondria *via* the import system, further stimulating Drp1 to promote fission, depleting MMP to cause Ca^2+^ efflux, cytochrome c release, endogenous apoptosis, and triggering p38 MAPK cascades to increase the inflammatory response (Ramachandran et al., 2020). VacA generated by *Helicobacter pylori* exacerbates apoptosis through similar stimulation, including the formation of anion-selective channels at the OMM to release cytochrome c into the cytosol and activate caspase 3 (Rassow, 2011). Host cell survival or death depends on mechanisms that are conducive to bacterial colonization. FimA, an effector protein produced by *Salmonella enterica*, maintains the relationship between VDAC1 and the glycolytic enzyme hexokinase to inhibit cytochrome c release by binding to VDAC1 at the OMM (Sukumaran et al., 2010). Another effector protein, SipB, facilitates autophagy-dependent cell death by disrupting the mitochondrial cristae morphology and imbalanced dynamics (Hernandez et al., 2003). In contrast, mitochondrial function also improves gut health and microbiota stability. In mice with increased mitochondrial activity, the relative abundance of *Dorea* and *Oscillospira* genera is enhanced and expression of SCFA, NAD, and sirtuin is elevated, leading to facilitation of fatty acid oxidation and reduction of metabolic disorders (Juárez-Fernández et al., 2022). Mitochondrial dysfunction in intestinal epithelial cells aggravates inflammation and oxygenation in the gut barrier and increases the abundance of facultative anaerobes, which are further involved in metabolic disorders (Zhang Y et al., 2022). Moreover, mitochondria also participate in the amelioration of lipid metabolism in the liver and reverse HFD-induced type 2 diabetes (Rodrigues et al., 2021). These data indicate that mitochondria outside vascular cells also respond to AS progression by regulating the gut microbiota. Therefore, gut bacteria not only regulate cell death by secreting toxins targeting mitochondria to promote bacterial colonization and reproduction but also activate the host’s innate immunity mediated by mitochondria to resist gut bacterial infections, subsequently leading to chronic inflammation and systemic diseases, including AS.

### 3.2 SCFA effects on mitochondria during AS

SCFAs are the most abundant type of gut microbiota metabolites derived from the colon and cecum of non-digestible carbohydrates, including acetate, butyrate, and propionate, which not only serve as ingredients for mitochondrial energy metabolism but are also involved in improving mitochondrial function (den Besten et al., 2013). Most SCFAs are absorbed by the host mainly through active transport mediated by translocated proteins in colonocytes, and the effects of SCFAs on mitochondria may depend on the SCFA concentration. Zhao et al. (Zhao T et al., 2020) found that butyrate mediates 54 genes associated with mitochondrial energy metabolism and exerts protective effects by increasing mtDNA and ATP contents under high insulin conditions. Butyrate also defends islet β cell activity *via* the depletion of mtROS and activation of antioxidant enzymes to inhibit oxidative and nitrosative stresses, lipogenesis, and loss of excess weight by stimulating GPR43 and beta-arrestin2 activity to favor mitochondrial biogenesis and energy expenditure, including triggering AMPK and the expression of PGC-1α and OXPHOS pathways, reducing the risk factors for AS (Tang et al., 2020). Administration of 3-hydroxybutyrate (3-HT) is beneficial and ameliorates atherogenesis (Zhang et al., 2021). Possibly, 3-HT binds to G-protein-coupled receptor 109a in macrophages to functionally facilitate cholesterol efflux and extracellular Ca^2+^ influx, leading to a decrease in the proportion of M1 macrophages *via* the suppression of NLRP3 and inactivation of ER stress *via* the inhibition of Ca^2+^ depletion (Zhang et al., 2021). The antioxidant response of butyrate significantly contributes to the induction of mitochondrial manganese–superoxide dismutase (MnSOD) and glutathione peroxidase expression by downregulating glycogen synthase kinase-3 beta and upregulating the nuclear translocation of nuclear factor erythroid 2-related factor 2 (Nrf2) (Tang et al., 2020). Propionic acid also regulates the redox state of the microenvironment through the mtROS content to influence chronic inflammatory diseases. Butyrate and its derivative butyramide attenuate insulin resistance and fat accumulation in the liver cells of obese mice by promoting mitochondrial fusion and the AMPK–acetyl–coenzyme A (CoA) carboxylase pathway to increase the utilization of fatty acids, glucose homeostasis, and mitochondrial respiratory rate (Mollica et al., 2017). SCFAs also suppress blood vessel cell death and chromatin modification by inactivating histone deacetylase, decreasing CD4^+^ and CD8^+^ T cell proliferation to inhibit vascular inflammation by delaying the maturation of dendritic cells, ultimately controlling AS progression (Andrade-Oliveira et al., 2015); therefore, SCFAs, especially butyrate, exert protective effects on mitochondria in vascular cells at a certain concentration (Table 1).

**TABLE 1:** Effects of intestinal microbiota metabolites on mitochondria and AS.

Metabolite	Subject	Effects on mitochondria	Effects on vessels	Ref
propionate	rats/Heart perfusions	increase glucose uptake and oxidation; promote mitochondrial CoA trapping and inhibit fatty acid oxidation	mediate perturbation of cardiac energy metabolism	Wang et al. (2018)
3–HB	ApoE^−/−^ mice	reduce the release of Ca^2+^ from the ER to mitochondria, inhibits ER stress	reduce systemic inflammatory and AS, increase cholesterol efflux	Zhang et al. (2021)
TMAO	patients with CAVD; AVICs	promote ER stress, mitochondrial stress, and NF–κB activation	increase aortic valve thicknesses	Li et al. (2022)
TMAO	HUVECs or aortas from ApoE^−/−^ mice	stimulate mtROS generation, inhibit SOD2 activation and SIRT3 expression	induce vascular inflammation and promote AS.	Chen et al. (2017)
TMAO	cardiac fibers	decrease LEAK and OXPHOS mitochondrial respiration with pyruvate and impair substrate flux *via* pyruvate dehydrogenase	induce disturbances in cardiac mitochondrial energy metabolism	Makrecka-Kuka et al. (2017)
TMAO	Wistar rats	prevent MCT–impaired mitochondrial energy metabolism by preserving fatty acid oxidation and decreasing pyruvate metabolism	restore right ventricular function, reduce heart failure severity, and maintain cardiac functionality	Videja et al. (2020)
TMAO	endothelial cells	modify the purinergic response affecting intracellular ATP–induced calcium increase, nitric oxide release, and eNOS^Ser1179^	impacts on endothelial eNOS phosphorylation	Querio et al. (2022)
TMAO	cardiac tissues from mice	significant reduction of phosphocreatine and ATP levels in cardiac tissue *via* suppression of mitochondrial complex IV activity	cause cardiac dysfunction with LV pressure overload	Yoo et al. (2022)
LPS	VSMC	stimulate PCSK9 release and induce mtDNA damage	cellular injury and apoptosis, promote AS	Ding et al. (2016)
LPS	HUVECs	decrease ATP content, MMP, and maximal respiration rate; increase expression of Drp1 with excessive mitochondrial fission	HUVECs injury	Lian N et al. (2022)
LPS	HUVECs	cause the mitochondrial permeability transition, cytosolic release of cytochrome c, and activation of caspases	induce apoptosis	Lim et al. (2009)
LPS	HUVECs	promote Drp1 phosphorylation, initiate the mitochondrial fission contributing to the caspase 9–dependent mitochondrial apoptosis	apoptosis of HUVEC	Cui et al. (2018)
H_2_S	ischemia–induced heart failure mice	increase ADP–stimulated oxygen consumption and ATP synthesis, elevate respiratory control ratios; attenuate oxidative stress	decrease infarct area/area at risk; decrease infarct area/LV.	Calvert et al. (2010)
H_2_S	aorta and VSMC	increase mtDNA–CN and mitochondrial maker gene expression, inhibit TFAM promoter methylation	regulate arterial tension	Li and Yang, (2015)
H_2_S	HUVEC	promote glucose uptake and ATP generation by glycolysis; shifts oxidative/glycolytic balance concomitant with inhibition of mitochondrial electron transport and OXPHOS.	trigger angiogenesis and improve vascular health	Longchamp et al. (2018)
H_2_S	H9c2 cardiomyocytes	increased ATP level and the expression of ATPase, decrease in ROS production and the enhancement in SOD, GPx, GST and SIRT1 expression	increase the cell viability and inhibit the cell apoptosis	Wu D et al. (2015)

Note: 3–HB, Ketone Body 3-Hydroxybutyrate; HUVEC, human umbilical vein endothelial cells; CAVD, calcified aortic valve disease; AVICs, human aortic valve interstitial cells; LEAK, substrate–dependent respiration; OXPHOS, oxidative phosphorylation; MCT, monocrotaline; PCSK9, proprotein convertase subtilisin/kexin type 9; LV, left ventricular.

### 3.3 Trimethylamine-N-oxide effects on mitochondria during AS

TMAO is a harmful bacterial metabolite associated with AS. After consuming dietary components containing carnitine, choline, and betaine, TMA derived from the gut microbiota is subsequently oxidized to TMAO *via* flavin-containing monooxygenases in the liver (Wang et al., 2011). After transplanting the cecal microbiota of C57BL/6J mice with a choline diet and high TMAO levels into germ-free ApoE^−/−^ mice, the recipients bore a greater atherosclerotic plaque burden and a positive association between TMAO levels in the plasma and atherosclerotic lesion area (Gregory et al., 2015). This indicates that choline diet-dependent TMAO levels may enhance atherosclerotic susceptibility. In a 3-year follow-up of 4,007 healthy participants with phosphatidylcholine challenges, the plasma levels of TMAO decreased with oral broad-spectrum antibiotics but increased after the withdrawal of antibiotics, which elevated the risk of major adverse cardiovascular events (MACE) (Tang et al., 2013), demonstrating the crucial role of gut microbiota in AS.

Mitochondria can be a target mediating TMAO effects on AS (Table 1). TMAO enhances aortic valve thickness and osteogenic differentiation by facilitating mitochondrial stress, including stimulation of mitochondrial swelling and NF-κB signaling (Li et al., 2022). TMAO also participates in the inhibition of mitochondrial respiration and OXPHOS. TMAO-treated cardiac fibers contribute to the decline in substrate-dependent respiration with pyruvate by 30% and negatively to β-oxidation, with declines in palmitoyl-CoA-related respiration and energy production (Makrecka-Kuka et al., 2017). TMAO also inhibits phosphocreatine and ATP levels by downregulating complex IV, further aggravating cardiac dysfunction and left ventricular pressure overload (Yoshida et al., 2022). Additionally, TMAO promote vascular endothelial injury by mitochondrial mediation. In ECs, TMAO administration increases succinate dehydrogenase complex subunit B overexpression, which promotes mtROS production and mitochondrial dysfunction, resulting in pyroptosis (Wu et al., 2020). TMAO upregulates mtROS levels and activates NLRP3 expression, likely by inhibiting SOD2 and SIRT3, further aggravating the inflammatory cascade in the blood vessels (Chen et al., 2017). TMAO mediates vascular cell adhesion molecule-1 expression in ECs to enhance monocyte adhesion, trigger scavenger receptors in macrophages, and recruit macrophages to promote the secretion of the inflammatory cytokines TNF-α and IL-6 (Mohammadi et al., 2016; Al-Obaide et al., 2017). In addition to increasing mtROS levels, TMAO-induced endothelial damage is strongly associated with other mitochondrial functions, such as the loss of MMP and Ca^2+^ influx, which affects NO release and eNOS phosphorylation (Querio et al., 2022). TMAO also induces Ca^2+^ and mtDNA release from platelets, causing dose-dependent platelet hyperreactivity and thrombus formation, which may be induced by ADP in the mitochondria (Querio et al., 2019). Moreover, TMAO might be reduced by the mitochondrial hmARC1.(Schneider et al., 2018). hmARC1 is an effective constituent of the mitochondrial amidoxime-reducing component protein that can translate TMAO into precursor TMA (Schneider et al., 2018). Overexpression of hmARC1 is correlated with the decline in TMAO in the liver as well as the enrichment of N-reductive activity in OMM. Collectively, mitochondria play a crucial role in mediating the effects of TMAO on AS progression, expanding the negative effects of TMAO on energy metabolism, oxidative stress, inflammatory cascade reactions, EC injury and death, and thrombus formation.

### 3.4 Hydrogen sulfide effects on mitochondria during AS

The bacterial metabolite H_2_S, which is generated by the gut microbiota or host cells and is the third physiologic signaling gas after CO and NO, shows dose-dependent effects on vessels, such as facilitating vasodilation and angiogenesis, decreasing monocyte adhesion, and attenuating apoptosis (Paul et al., 2021). The aorta has the highest H_2_S bioavailability and most H_2_S bioequivalents (Shen et al., 2013). H_2_S triggers cGMP synthesis, inhibits the degradation of cGMP and cAMP to promote NO release and vasodilatation, and activates NAD+ in ECs to delay endothelial senescence. Moreover, H_2_S has a pro-angiogenic effect partly through the regulation of electron transport, OXPHOS, and sulfhydration with mitochondria to elevate glucose uptake and glycolysis (Longchamp et al., 2018).

As H_2_S is sensitive to oxygen, the metabolic site of H_2_S is mainly in the mitochondria. The anaerobic microbiota generates a non-negligible amount of H_2_S in the colon, which is oxidized into detoxicated thiosulfate and sulfate mediated by mitochondria and excreted. The released electrons can be transferred to the electron transport chain (ETC), further improving ATP production during this metabolic process (Libiad et al., 2019). H_2_S reduces oxidative stress and mitochondrial swelling while enhancing mitophagy and mitochondrial biogenesis and mediates the sulfidation of the mitophagy protein parkin, further activating E3 ubiquitin ligase and inducing mitophagy (Sun et al., 2020). It also improves mitochondrial biogenesis in myocardial cells following ischemia/reperfusion (I/R) injury by downregulating protein phosphatase 2A and upregulating peroxisome PGC-1α (Calvert et al., 2010; Untereiner et al., 2016). In H_2_O_2_-treated cardiomyocytes, NaHS effectively attenuates mtROS production, reduces damage from oxidative stress, and enhances cardiomyocyte viability and activity *via* a SIRT1-dependent pathway (Wu D et al., 2015). SIRT1 is a significant element in mediating the effects of NaHS on reversing oxidative stress and depleting ATP. These effects can be eliminated by SIRT1 silencing (Ma et al., 2019). Furthermore, NaHS ameliorates EC senescence by upregulating SIRT1 and antioxidant defenses (Zheng et al., 2014). Low doses of H_2_S are beneficial to cells as they promote mitochondrial biological function and ameliorate apoptosis, whereas high concentrations inactivate complex III at the ETC and even lead to apoptosis of aortic SMCs (Murphy et al., 2019). Excess H_2_S is cytotoxic and harmful to cellular energy metabolism and causes DNA injury through the inhibition of mitochondrial respiration and ATP synthesis (Borisov and Forte, 2021). High concentrations of H_2_S bind to the copper center of cytochrome c oxidase to inhibit its activation and suppress mitochondrial activity (Módis et al., 2014). This further confirms that mitochondria play a crucial role in regulating the interaction between H_2_S from the gut microbiota and vascular damage (Table 1).

### 3.5 Other intestinal flora metabolites

**FIGURE 2:**
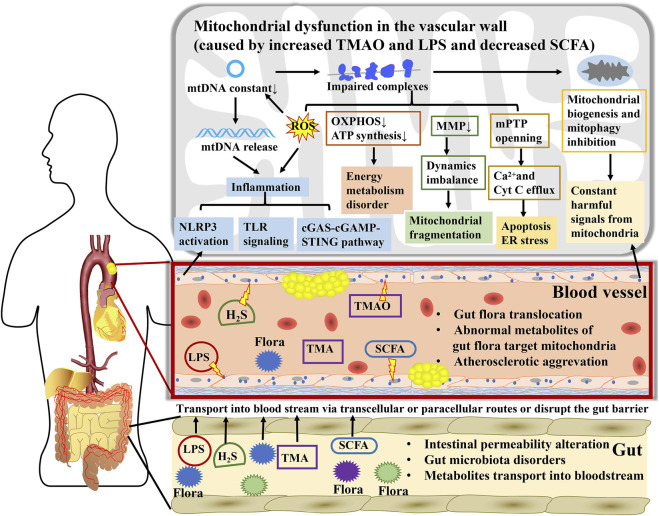
A key mediator linking gut microbiota to atherosclerosis, mitochondria participate in the interaction between numerous metabolites from gut microbiota and atherosclerosis. During atherosclerosis, gut microbiota disorders and intestinal permeability alteration contribute to direct transposition of flora to arteries. Microbial metabolites are transported into the bloodstream *via* transcellular or paracellular routes or disrupting the gut barrier and subsequently affect mitochondrial function in blood vessels cells. Mitochondria expand the influence of TMAO, LPS, H_2_S, and SCFA on energy metabolism, oxidative stress, inflammatory cascade reaction, and endothelial cell injury through regulating mtDNA release, ATP synthesis, MMP, mPTP, mitochondrial biogenesis, and mitophagy, further promoting or opposing the atherogenic process.

Other metabolites from the gut microbiota also correlate with the development of AS (Table 1). Small metabolites are transported into the bloodstream *via* transcellular or paracellular routes, whereas large metabolites disrupt the gut barrier and are released into the body (Kirichenko et al., 2020). Indole derivatives, such as indoxyl sulfate, indole ethanol, and indole acrylic acid, are produced by the gut microbiota from tryptophan and induce proinflammatory activity after entering the bloodstream (Xu H et al., 2022). In addition, indolepropionic acid (IPA), which is metabolized from tryptophan, is substantially reduced in patients with coronary artery disease. Oral IPA administration mitigates the extent of atherosclerotic lesions by activating ABCA1 and promoting RCT (Xue et al., 2022). Another amino acid derivative metabolized by the gut microbiota is phenylacetylglutamine, which induces platelet hyper-responsiveness and thrombotic action (Liu et al., 2021) and is positively associated with MACE (Bogiatzi et al., 2018). Furthermore, secondary bile acids (BAs) and LPS are microbiota-dependent proatherogenic molecules. BAs are converted by the gut microbiota from primary BAs and further mediate lipid and glucose metabolic pathways by binding to receptor farnesoid X and membrane receptor Takeda G-protein-coupled receptor 5, resulting in the development of vascular inflammation, lipid deposition, and AS (Pols et al., 2011; Kuipers et al., 2014). Indole-3-carboxylic acid and BAs contribute to mitochondrial swelling and increase mitochondrial permeability by changing the Ca^2+^ concentration threshold for mPTP opening (Fedotcheva et al., 2021). Binding of secondary BAs to the G-coupled membrane protein 5 and farnesoid X receptor triggers mitochondrial fission *via* the ERK/DRP1 pathway and mediates fatty acid uptake and β-oxidation, further causing energy metabolism disorders and apoptosis (Mikó et al., 2018). LPS, a large molecule derived from Gram-negative bacteria, translocates into the bloodstream by compromising the intestinal mucosa, leading to metabolic endotoxemia (Giusti et al., 2017). LPS subsequently stimulates the TLR 4 and TLR4/MyD88/TRIF signaling pathways to trigger macrophages and systemic inflammation (Caesar et al., 2015). Additionally, some Gram-negative bacteria can translocate to the arterial intima and directly release LPS, leading to AS progression (Verhaar et al., 2020). Consequently, the gut microbiota–mitochondria axis acts as a significant endocrine metabolic pathway to regulate the host’s systemic chronic diseases, especially AS. The specific mechanism might also be affected by the dominant bacteria in the gut, concentration of metabolites, and other atherosclerotic risk factors. Further studies are necessary to determine the long-distance interactions between the gut microbiota, mitochondria, and AS completely and accurately **(** Figure 2 **).**

## 4 Natural compounds in herbal medicine regulating intestinal flora and mitochondria to treat AS

Intervention of gut microbiota and mitochondria is a novel therapeutic target for AS. Lifestyle adjustments may simultaneously regulate gut microbiota and mitochondria (Najjar and Feresin, 2019). Endurance exercise maintains the stability of intestinal flora and reverses host mitochondrial functions by inhibiting enteric inflammation and diminishing the reproduction of pathobionts in the intestinal tract (Clark and Mach, 2017). Plant-based diets or consumption of unsaturated fatty acids can improve gut microbiota diversity and symbiotic homeostasis and recover mitochondrial respiration and fatty acid oxidation (López-Salazar et al., 2021), resulting in enhanced satiety, increased energy expenditure, and elevated insulin sensitivity, ultimately ameliorating AS by reducing body fat and maintaining glycemic stability. However, the therapeutic effects of exercise and dietary changes are limited. Herbal medicine has been used to treat CVDs in China for many years because of the advantages of multi-target, synergistic, and systemic therapy. Herbs are closely related to gut microbiota and mitochondria (Zhou et al., 2022). Gut microbiota converts herbs into active metabolites with higher bioavailability, and herbs regulate gut microbiota biodiversity to ameliorate CVD in the host, similar to prebiotics **(** Table 2 **)** (Ma et al., 2022; Qi et al., 2022).

**TABLE 2:** Effects of natural compounds from herbs on gut flora, mitochondria, and AS.

Natural compound	Model	Administration	Effects on gut flora/mitochondria	Effects on vessels	Ref
BBR	HFD–fed Apoe^−/−^ mice	0.5 g/L in drinking water for 14w	increase the abundance of Akkermansia spp; increase intestinal expression of tight junction proteins and the thickness of the colonic mucus layer	decrease arterial and intestinal expression of proinflammatory cytokines and chemokines; have antiatherosclerotic and metabolic protective effects	Zhu L et al. (2018)
BBR	HFD–fed Apoe^−/−^ mice	50 mg/kg twice weekly by gavage; FMT	increase Firmicutes and Verrucomicrobia; decrease Bacteroidetes and Proteobacteria; reduce TMAO level and FMO3 expression	reduce collagen content in atherosclerotic plaque and MMP–2, IL–6 and ICAM–1 expression	Shi Y et al. (2018)
BBR	Ang II– infused C57BL/6 J mice	150 mg/kg/d for 4 w	reduce the Firmicutes/Bacteroidetes ratio and increase the abundances of *Lactobacillus* and plasma TMA/TMAO production; inhibit FMO3 expression	alleviate the elevated blood pressure, vascular dysfunction, and vascular pathological remodeling	Wang Z et al. (2022)
BBR	choline– fed ApoE^−/−^ mice	200 mg/kg/d for 6w	attenuate TMA/TMAO production and abundance of CutC and CntA genes; inhibit the choline–to–TMA transformation in the strains of C. sporogenes and A. hydrogenalis	mitigate atherosclerotic lesion areas	Li X et al. (2021)
BBR	HFD–fed Apoe^−/−^ mice	50 mg/kg/d by gavage	adjust the composition of intestinal flora; elevate lipid and glycan metabolism and synthesis of SCFAs; reduce TMAO production	decrease pro–inflammatory cytokines, lipid level in plasma, and atherosclerotic lesions	Wu et al. (2020)
BBR	HFD–fed hamsters	100 mg/kg orally; FMT	reduce the TMA production in the *E. coli* and Eubacterium and TMAO in plasma and faecal samples; suppress FMOs activity; adjust the composition of intestinal flora	decrease blood glucose and lipids; reduce development of plaques in AS patients	Ma et al. (2022)
BBR	AS patients	orally 1 g/d for 4m	decline of Eubacterium_hallii_group, Anaerostipes, Faecalibacterium, Dialister, Eubacterium_coprostanoligenes_group, Coprococcus_3, Butyricicoccus and *Clostridium*_sensu_strito_1		Ma et al. (2022)
BBR	TAC–induced chronic HF	50 mg/kg/d for 4w by gavage	upregulate PINK1/Parkin–mediated mitophagy	ameliorate cardiac dysfunction, cardiac hypertrophy, interstitial fibrosis, and cardiomyocyte apoptosis	Abudureyimu et al. (2020)
BBR	MI/R; H9C2 cells	300 mg/kg/d for 3d	increase MMP; regulate autophagy–related protein expression; induce cell proliferation and autophagosome formation	reduce the myocardial infarct size, cardiomyocyte apoptosis, and the expression of myocardial enzyme (CK–MB, LDH, and AST)	Zhu et al. (2020)
RSV	HFHSD–fed C57Bl/6N mice	chow with 0.4% RSV; FMT for 8w	decrease relative abundance of Turicibacteraceae, Moryella, Lachnospiraceae, and Akkermansia; increase relative abundance of *Bacteroides* and Parabacteroides	improve glucose homeostasis in obese mice	Sung et al. (2017)
RSV	HFHSD–fed C57Bl/6N mice	FMT	few changes in fecal metabolites and the metabolic profile; reduce inflammatory cytokine level	decrease inflammation in the colon; reduce the systolic blood pressure of hypertensive mice	Kim et al. (2018)
RSV	Choline– or TMA–fed in mice	chow with 0.4% RSV	increase Bacteroidetes; decrease Firmicutes; reduce TMAO synthesis and ileal BA content; enhance BA deconjugation and fecal excretion; repress the enterohepatic FXR– FGF15 axis; increase CYP7A1 expression and hepatic BA neosynthesis	decrease the atherosclerotic lesion area and the cholesterol content in the whole aorta	Chen M L et al. (2016)
RSV	t–BHP–induced HUVECs	0.1, 1, 10, 15 μM 2h	repress collapse of MMP and mtROS generation; increase enzymatic activities of IDH2, GSH–Px and SOD2	increase cell viability; inhibit cell apoptosis	Yang et al. (2019)
RSV	PA–induced HUVECs	10 μM for 8 h	improve expression of MFN1, MFN2, and OPA1; inhibit fragmentation of mitochondria; reduce oxidative stress	attenuate endothelial oxidative injury	Zhou et al. (2014)
RSV	ox–LDL induced HUVEC	5, 10, 20, 40, 80, 120 μg/ml 24 h	increase MMP, Bcl–2/BAX ratio and SOD2; decrease the release of mitochondrial cytochrome c into the cytoplasm; reduce the activation of caspase and lipid peroxidation	possess protective effects against endothelial cell apoptosis and oxidative damage	Liu et al. (2017)
RSV	older glu–intolerant adults	2–3 g/d for 6 w	increase OXPHOS perturbed pathways and mitochondrial content	have beneficial effects on vascular function	Pollack et al. (2017)
allicin	ʟ–carnitine –fed ApoE^−/−^ mice	orally 10 mg/kg/d in 0.5% CMC	inhibit γBB and TMA; reduce serum d9–TMA and d9–TMAO; inhibit microbial carnitine→γBB→TMA pathways	reduce aortic plaques	Panyod et al. (2022)
allicin	OCCT in Nine volunteers	garlic: water (ratio 1:3) were mixed	reduce TMAO formation; improve gut microbial diversity; increase the relative abundances of beneficial bacteria; increase γBB; prevent the microbial transformation of γBB into TMA	reduction in platelet aggregation	Panyod et al. (2022)
allicin	LPS–induced HUVECs	0–40 μg/ml 24 h	decrease the MMP collapse, cytochrome c synthesis and mitochondrial ATP release; suppress ROS overproduction; reduce lipid peroxidation the endogenous antioxidant enzyme activities	enhance HUVEC proliferation; ameliorate apoptosis	Zhang M et al. (2017)
allicin	HFD–fed C57BL/6 mice	100 mg/kg/d	increase the abundance of Bacteroidales and Clostridiales at the order level; increase the abundance of Akkermansia at the genus level	improve lipolysis and insulin sensitivity by suppressing hepatic lipid synthesis and increasing thermogenesis	Shi et al. (2019)
curcumin	HFD–fed Apoe^−/−^ mice	0.1% for 16w	decrease intestinal cholesterol absorption	reduce the extent of atherosclerotic lesions by 45%; reduce cholesterol accumulation in the aortas by 56%	Zou et al. (2018)
curcumin	Aging mice	dietary 0.2%	promote eNOS and AMPK phosphorylation; upregulate UCP2 and reduced ROS production	improve cerebrovascular endothelium–dependent relaxation in aging	Pu et al. (2013)
THC	MI/R injury	50 mg/kg/day	increase SOD and CAT activities; decrease MDA level; enhance MMP; diminish the Bax/Bcl–2 ratio and cleave caspase–3 level	increase EF and FS; decrease LVESD and LVESV	Chen et al. (2021)
QUE	HCD–fed ApoE^−/− ^mice	100 mg/kg/d. for 12w	decrease the abundance of Phascolarctobacterium and Anaerovibrio genera	protect damaged vessels; exhibit a thinner intima hyperplasia; less inflammatory cells	Wu et al. (2019)
QUE	HUVECs	10 µM 1 h	depolarize the mitochondrial membrane; increase level of the mitoBK_Ca_ β3 regulatory subunit	enhance cellular migration; beneficial for vascular endothelial cells	Kampa et al. (2021)
QUE	50 μM iron	20 μM for 48 h	decrease mitochondrial oxidative stress and mPTP opening, increase mitochondrial membrane potential	attenuate iron overload induced HUVECs mitochondrial dysfunction	Chen et al. (2020)
QUE	Ldlr^−/−^ mice	100 ug/d in an aqueous solution containing 1% sodium lauryl sulphate	reduce the abundance of Verrocomicrobia; increase microbiome diversity and the abundances of Actinobacteria, Cyanobacteria and Firmicutes	reduce the extent of atherosclerotic lesions in the aortic sinus	Nie et al. (2019)
QUE	Pi–induce VSMCs	50 μM 6 d	inhibit oxidative stress; decrease mitochondrial fission by inhibiting the expression and phosphorylation of Drp1	block apoptosis and calcification of VSMCs; ameliorate aortic calcification	Cui et al. (2017)
QUE	apoE^−/−^ mice	20 mg/kg/d 8 w	increase MMP; decrease ROS generation	decrease lipid deposition in arterial lumina, serum sIcam–1, and IL–6 and Vcam–1 in aorta, while increase the density of SIRT1 in aorta	Jiang et al. (2020)
QUE	ox–LDL–induced HAECs	0.3, 1, or 3 μM 48 h		decrease the expression of SaβG; improve cell morphology	

Note: BBR, berberine; RSV, resveratrol; Que, Quercetin; HFD, high–fat diet; HCD, high cholesterol diet; Ang II, Angiotensin II; TAC, transverse aortic contraction; HF, heart failure; HFHSD, high fat/high sugar; t–BHP, tert–butyl hydroperoxide; HUVECs, human umbilical vein endothelial cells; PA, palmitic acid; OCCT, oral carnitine challenge test; MI/R, myocardial ischemia–reperfusion; Ldlr^−/−^.

low–density lipoprotein receptor–null; Pi, inorganic phosphate; THC, tetrahydrocurcumin; FGF15, fibroblast growth factor 15; CYP7A1, cholesterol 7a–hydroxylase; IDH2, isocitrate dehydrogenase 2; γBB, γ–butyrobetaine; SaβG, senescence–associated β–galactosidase; FMT, fecal microbiota transplantation; CutC, enzyme/co–enzyme of choline–trimethylamine lyase; MDA, malondialdehyde; EF, ejection fraction; FS, fractional shortening; LVESD, left ventricular end systolic diameter; LVESV, left ventricular end systolic volume; mitoBK_Ca_, mitochondrial large–conductance Ca^2+^–regulated potassium; IDH, isocitrate dehydrogenase; CK–MB, creatine kinase–MB; LDH, lactate dehydrogenase; AST, aspartate aminotransferase; HF, heart failure; TAC, transverse aortic contraction; h, hours; d, days; w, weeks; m, months.

### 4.1 Berberine

Berberine (BBR) is an isoquinoline alkaloid found mainly in *Coptis chinensis* and *Berberis pruinosa var. pruinosa* (Habtemariam, 2020). BBR has low oral bioavailability and absorption owing to its poor solubility. Modulating the gut microbiota to some extent increases the efficiency of BBR in CVDs. BBR effectively mitigates AS in high-fat diet-fed ApoE^−/−^ mice by upregulating the abundance of intestinal *Akkermansia* (Zhu L et al., 2018), *Verrucomicrobia, Firmicutes* (Shi Y et al., 2018), *Alistipes,* and *Roseburia* (Wu et al., 2020), while downregulating that of Lachnospiraceae*, Bacteroidales,* and *Eubacterium*. Changes in the intestinal microbiota recover gut barrier integrity, affect serum TMAO (Shi Y et al., 2018), secondary BAs, and SCFA levels, and attenuate proinflammatory cytokines, chemokines, total cholesterol, and very low-density lipoprotein (Wu et al., 2020), thereby reversing atherosclerotic lesions. BBR is metabolized to dihydroberberine by the gut microbiota (Ma et al., 2022), and dihydroberberine further inactivates the enzymes choline-trimethylamine lyase and flavin monooxygenase, enhancing the diversity of the microbiome to suppress TMAO production and decreasing atherosclerotic lesion (Li X et al., 2021). Moreover, BBR ameliorates the risk factors for AS, including obesity (Hu et al., 2012), hyperglycemia (Li M et al., 2020), and hyperlipidemia (Yang et al., 2022), by maintaining the balance of intestinal flora.

BBR might target mitochondria to exert a protective effect on AS mediated by the gut microbiota. BBR protects ECs from oxidative stress by inhibiting Nox4 protein and ROS expression to improve vasodilation (Cheng et al., 2013). Nox4 in vessels is mainly distributed in the mitochondrial membrane and is involved in stimulating mtROS production. A decline in mtROS caused by BBR further elicits a lower expression of the NLRP3 inflammasome and increases MMP. BBR greatly improves mitophagy by preserving myocardial function and suppressing apoptosis. For example, BBR improves cardiac hypertrophy and cardiomyocyte apoptosis *via* the PINK1/parkin pathway (Abudureyimu et al., 2020), induces mitophagy to inhibit NLRP3 activation by reducing P62 and recruiting LC3 to mitochondria (Liu H et al., 2020), restores cardiomyocyte senescence by activating prohibitin 2 (Wang L et al., 2022), and defends the myocardium from I/R injury *via* the hypoxia-induced factor (HIF)-1α/BCL2-interacting protein 3(BNIP3) pathway (Zhu et al., 2020). Furthermore, BBR also decreases basal oxygen consumption rates and facilitates fatty acid oxidation by diminishing mitochondrial swelling and upregulating mitochondrial biogenesis mediated by the AMPK/PGC-1α pathway to attenuate lipid deposition and weight in obese mice (Yao et al., 2020; Yu et al., 2021). BBR exerts a protective effect on fatty acid oxidation enzymes, mitochondrial dynamics balance, and ATP synthesis in cells induced by high glucose (Rong et al., 2021). Collectively, BBR remarkably recovered the gut flora and metabolic balance and preserved mitochondrial function, contributing to the inhibition of AS progression.

### 4.2 Resveratrol

Resveratrol (RSV) is a plant-derived polyphenol extracted from grapes, peanuts, blueberries, and mulberries and has been used as a potential prebiotic in the intervention of AS. RSV attenuates atherosclerotic plaques by enriching *Bacteroides, Lactobacillus, Parabacteroides,* and *Bifidobacterium* (Chen M L et al., 2016; Sung et al., 2017), reducing TMAO levels and increasing BA excretion. However, a combination of antibiotics and RSV cannot suppress TMAO or mitigate AS. These results revealed the important role of the gut microbiota in the anti-atherosclerosis treatment of RSV (Chen M L et al., 2016). RSV also mitigates insulin resistance and triglyceride deposition in the liver to suppress the effects of metabolic syndrome on AS by increasing the proportion of *Akkermansia spp.* in the intestine (Anhê et al., 2015). Fecal transplantation following resveratrol treatment in mice is beneficial for improving glucose metabolism and low-grade inflammation in obese mice (Sung et al., 2017). In turn, the gut microbiota converts resveratrol into its derivatives to enhance bioavailability, such as dihydroresveratrol, 3,4′-dihydroxy-trans-stilbene and 3,4′-dihydroxybibenzyl (Bode et al., 2013), which is the predominant form in the plasma and effectively protects ECs and VSMCs.

RSV induces endothelial relaxation by activating eNOS and NO synthesis, diminishing ET-1, and inhibits VSMCs proliferation and senescence by decreasing β-galactosidase and pro-fibrotic proteins to ameliorate vascular remodeling and sclerosis (Kim et al., 2018). Possible mechanisms are closely related to mitochondrial function. In human coronary arterial ECs, RSV exerts protective effects on mitochondrial biogenesis by upregulating PGC-1α, Nrf-1, and TFAM, and triggering eNOS expression and NO release by inducing SIRT1 (Csiszar et al., 2009; Davinelli et al., 2013). RSV also improves complex I and III expression in the ETC and BNIP3-related mitophagy by triggering HIF1 and AMPK, leading to mitochondrial redox balance and mitochondrial renewal in ox-LDL-induced ECs (Li C et al., 2020). RSV is well-known for its antioxidant properties. It ameliorates mitochondrial fragmentation to diminish mtROS levels by enhancing Mfn1/2 and OPA1 activation (Yang et al., 2019). RSV significantly decreases mtROS levels by activating AMPK-PGC-1α-ERRα-SIRT3 signaling (Zhou et al., 2014) and inhibiting antioxidant enzymes to defend EC activity from H_2_O_2_ and deplete apoptosis by attenuating cytochrome c efflux from the mitochondria and Bcl-2 expression (Liu et al., 2013; Liu et al., 2017). SIRT1 is also involved in the antioxidant activity of RSV. RSV is a well-known activator of SIRT1 (Csiszar et al., 2008; Wellman et al., 2017) that can stimulate MnSOD and glutathione (GSH) to reduce the levels of O_2_^•−^and H_2_O_2_ in the endothelial mitochondria (Ungvari et al., 2009). In clinical trials, RSV-treated older adults with glucose intolerance exhibited symptoms of an enhanced fasting reactive hyperemia index, showing a restoration of endothelial function, probably caused by increasing mitochondrial content and facilitating OXPHOS (Pollack et al., 2017). Consequently, RSV can simultaneously improve endothelial injury, vascular thickening, and fibrosis by modulating intestinal microbiota homeostasis and the mitochondrial redox balance.

### 4.3 Allicin

Allicin is a thioester of sulfenic acid isolated from *Allium sativum L.* [Amaryllidaceae] and effectively crosses cell membranes to play antibacterial and antioxidant roles owing to its hydrophobic characteristics. Garlic is a recognized prebiotic; thus, allicin can be metabolized into diallyl disulfide (DAD) and diallyl trisulfide (DAT) to regulate gut microbiota composition and disrupt TMA production in ApoE^−/−^ mice with high concentrations of carnitine (Panyod et al., 2022). Following allicin treatment, the proportions of *Bifidobacterium, Lactobacillus, Bacteroidales,* and *Clostridiales* significantly increased in the gut (Shi et al., 2019), while those in some pathogenic bacteria, such as *Proteobacteria, Escherichia–Shigella,* and *Streptococcus,* drastically reduced (Hu et al., 2021). Moreover, allicin also regulates the metabolites of gut microbiota, such as upregulating SCFA, while downregulating secondary BAs, TMAO, and indoles, stimulating brown adipose tissue activation, browning in white adipose tissues (Zhang et al., 2020), decline in lipid accumulation in the liver, and weight loss in obese mice (Shi et al., 2019).

Allicin can recover mitochondrial function in ECs and ameliorate AS. DAT contributes to protecting ECs from oxidative stress under high-glucose conditions by facilitating mitochondrial respiration, triggering MnSOD and glutathione peroxidase (GSH-Px) expression, and suppressing lipid peroxidation (Liu et al., 2014). Moreover, allicin is involved in elevating MMP and inhibiting cytochrome c release and ATP export as a DAMP by inactivating antioxidant enzymes and stimulating Nrf2 activation, resulting in the reversal of endothelial apoptosis and vascular inflammation (Zhang M et al., 2017). DAD can prevent cardiac hypertrophy by improving mitochondrial biogenesis, including activation of the mitochondrial complex and the eNOS–Nrf2–TFAM signaling pathway (Khatua et al., 2016). This demonstrated the antibacterial and antioxidant properties of allicin. Allicin, DAD, and DAT are the main probiotic compounds that preserve cardiovascular diastolic function and cell activity by resisting mitochondria-related oxidative stress.

### 4.4 Quercetin

Quercetin (Que) is a flavonoid naturally found in various vegetables, fruits, nuts, tea, and wine. Que has become an increasingly popular natural dietary supplement owing to its association with CVDs. The anti-atherosclerotic effects of Que in maintaining intestinal homeostasis have been widely reported. Oral administration of Que reduces lipid metabolites, such as atherogenic lysophosphatidyl choline, in the plasma and artery wall, which are negatively correlated with the abundance of *Actinobacteria, Firmicutes,* and *Cyanobacteria* in the gut (Nie et al., 2019). Que notably diminishes plasma lipid levels (TC, TG, HDL, and LDL) and plasma inflammation (TNF-α and IL-6) through the BA biosynthesis pathway metabolized by the gut microbiota with a KEGG analysis (Wu et al., 2019). Que also prevents lipid accumulation in vascular macrophages and elevates foam cell survival rates by inducing autophagy and oxidative resistance (Cao et al., 2019). Que is involved in many aspects of lipid metabolism, from the gut to vessels, to attenuate AS.

Que plays a protective role by increasing mitochondria-related cellular viability. Que opens the mitochondrial large-conductance Ca^2+^-regulated potassium channel, leading to MMP depolarization and maintenance of EC environmental homeostasis (Kampa et al., 2021). Alterations in MMP and permeability caused by oxidative damage could contribute to apoptosis and direct damage to NO content, while Que could reverse these phenomena to preserve EC activity induced by iron or angiotensin II (Lu et al., 2016; Chen et al., 2020). Que also has potential benefits against mitochondrial fragmentation mediated by Drp1 overexpression, which enhances mtROS levels and exacerbates VSMCs apoptosis *via* cytochrome c release and caspase-3 activation, further causing vascular calcification (Cui et al., 2017). The mitochondrial protective effects have also been confirmed *in vivo*. Que remarkably mitigated the extent of atherosclerotic plaque area and decreased lipid deposition in the aorta and soluble intercellular adhesion molecule-1 in ApoE^−/−^ mice, probably because of increased MMP and decreased mtROS levels to resist endothelial senescence and apoptosis (Jiang et al., 2020). Briefly, the cardiovascular targets of Que treatment have been established along the gut microbiota–mitochondrial axis.

### 4.5 Curcumin

Curcumin, a bioactive polyphenolic of *Curcuma longa L.* [Zingiberaceae], is involved in anti-atherosclerosis by remodeling gut microbiota dysbiosis, altering the level of metabolites, and cytoprotection targeting mitochondria. Curcumin can reduce TMAO levels to diminish atherosclerotic lesions induced by cadmium exposure (Zhang J et al., 2022), probably by increasing bacterial richness (McFadden et al., 2015) and improving the defense of the intestinal barrier (Wang et al., 2017). Effective regulation of intestinal microbiota using curcumin can further inhibit the absorption of intestinal cholesterol (Hong et al., 2022) and mitigate low-grade systemic inflammation (Cox et al., 2022), including a 56% reduction in cholesterol deposition in the aorta (Zou et al., 2018) and a decline in monocyte chemoattractant protein 1 (Zhou et al., 2021), lipocalin 2 (Wan et al., 2016), TNFα, NFκB, and C-reactive protein expression. The therapeutic effect of curcumin on AS also depends on improving bioavailability mediated by the gut microbiota (Lopresti, 2018), further strengthening its positive influence on the distal mitochondria. Curcumin protects ECs and cardiomyocytes from mitochondrial oxidative stress and MMP loss (Gupta et al., 2016; Chen et al., 2021). Specifically, curcumin can upregulate UCP2 to reduce the generation of downstream mtROS, leading to enhanced eNOS and AMPK phosphorylation and recovery of endothelial functions (Gupta et al., 2016). Curcumin inhibits cardiomyocyte autophagy and apoptosis induced by hypoxia and reoxygenation injury by facilitating the expression of antioxidant enzymes, increasing MMP, and suppressing mitochondria-related apoptosis (Chen et al., 2021). Therefore, curcumin acts as an anti-atherogenic agent by ameliorating the intestinal flora and mitochondria to prevent lipid accumulation, inflammation, and oxidative stress.

There are multiple other bioactive compounds extracted from herbal medicines, including ginkgolide B (Lv et al., 2021), pterostilbene (Zhang et al., 2012), and catalpol (Zhang Y et al., 2018), which are beneficial for maintaining the connection between the bacterial flora, metabolites, and mitochondria and contribute to the recovery of vascular stability and inhibition of AS progression. Thus, herbal medicines and their natural compounds can sustain the balance between the microbiota and the host. However, further research is necessary to reveal the therapeutic effects of herbal medicines on the immune and metabolic networks between gut microbiota and mitochondria.

## 5 Conclusion

Although the risk factors and therapeutic methods for AS have been constantly improving, atherosclerotic CVD remains the leading cause of death worldwide. This review focuses on the close association between gut microbiota and mitochondria during AS progression and simultaneously enumerates several common herbal medicines and their natural compounds to prevent AS by adjusting the intestinal flora and sustaining mitochondrial function. Derived from the endosymbiotic α-proteobacterium, mitochondria possess characteristics similar to those of bacteria, such as exporting DAMP to activate innate immunity, which makes mitochondria a target for the gut microbiota and its metabolites, including SCFA, TMAO, and H_2_S. Mitochondria not only participate in providing energy but also act as activators of cholesterol metabolism, inflammation, and apoptosis, facilitating the development of AS. Herbal medicines are widely used in China owing to their advantages of multi-target and systemic treatments, which enable the simultaneous regulation of the intestinal flora and vascular mitochondria. Gut microbiota have not only been shown to be endocrine organs that regulate metabolism but are also significantly responsible for reversing the poor bioavailability of natural compounds. Importantly, the interactions between the gut microbiota and mitochondria collectively establish an immune and metabolic network that influences AS progression, which could be a potential target of herbal medicine to prevent AS. However, the causal relationships among microbiota, mitochondria, and AS remain unclear. The safety of herbs, including safe dosage, treatment duration, and potential side effects, has not been investigated in detail. Subsequent analyses are required to systematically elucidate a complete and accurate picture of gut microbiota, metabolites, mitochondria, and local atherosclerotic changes *via* metagenomic, metabolomic, and cytological methods.
